# Consistency across Repeated Eyewitness Interviews: Contrasting Police Detectives’ Beliefs with Actual Eyewitness Performance

**DOI:** 10.1371/journal.pone.0118641

**Published:** 2015-02-19

**Authors:** Alana C. Krix, Melanie Sauerland, Clemens Lorei, Imke Rispens

**Affiliations:** 1 Maastricht University, Forensic Psychology Section, Maastricht, The Netherlands; 2 University of Applied Sciences for Police and Administration of Hesse, Wiesbaden, Germany; 3 Police Academy of the Netherlands, Apeldoorn, The Netherlands; University of Akron, UNITED STATES

## Abstract

In the legal system, inconsistencies in eyewitness accounts are often used to discredit witnesses’ credibility. This is at odds with research findings showing that witnesses frequently report reminiscent details (details previously unrecalled) at an accuracy rate that is nearly as high as for consistently recalled information. The present study sought to put the validity of beliefs about recall consistency to a test by directly comparing them with actual memory performance in two recall attempts. All participants watched a film of a staged theft. Subsequently, the memory group (*N* = 84) provided one statement immediately after the film (either with the Self-Administered Interview or free recall) and one after a one-week delay. The estimation group (*N* = 81) consisting of experienced police detectives estimated the recall performance of the memory group. The results showed that actual recall performance was consistently underestimated. Also, a sharp decline of memory performance between recall attempts was assumed by the estimation group whereas actual accuracy remained stable. While reminiscent details were almost as accurate as consistent details, they were estimated to be much less accurate than consistent information and as inaccurate as direct contradictions. The police detectives expressed a great concern that reminiscence was the result of suggestive external influences. In conclusion, it seems that experienced police detectives hold many implicit beliefs about recall consistency that do not correspond with actual recall performance. Recommendations for police trainings are provided. These aim at fostering a differentiated view on eyewitness performance and the inclusion of more comprehensive classes on human memory structure.

## Introduction

Imagine a witness who is interviewed by the police at the crime scene. He describes the perpetrator as middle-aged, slim, brown-haired, and wearing jeans. Several weeks later, the witness is invited to a second police interview after being identified as a key witness. Here, he provides another perpetrator description that is mostly consistent with the earlier description. However, he now remembers the perpetrator to be blonde (instead of brown-haired) and comes up with new information, that is, a black cardigan. In fact, witnesses are likely to be interviewed several times during the investigations [1] and consequently, it is possible that contradictory or new details are recalled. Chances are that the witness of this example will not be considered very credible in court, as jurors are explicitly instructed to attend to inconsistencies in the statements to determine the witness’ credibility [2]. Indeed, inconsistent statements are regarded as an indicator of overall inaccuracy by police officers, lawyers, and mock-jurors alike [3–5]. This belief, however, is in direct contrast to research findings showing that (in)consistency of recall is not or only weakly related to overall recall accuracy [3,6–8].

When it comes to inconsistencies, it is important to distinguish the different types of inconsistency. While direct contradictions generally imply that at least one of the reported details is incorrect, this is not applicable to reminiscent details (i.e., details that are recalled only in a later, but not in the first interview). Even though reminiscence seems at odds with the idea of declining memory over time, previous research has shown that it is a widespread phenomenon and that forgetting does not preclude reminiscence [9–11]. In fact, when recalling twice about the same incident, almost all participants exhibit reminiscence [12–14] and accuracy rates of reminiscent details can be quite high. Although the accuracy of the latter may not always be as high as the accuracy of consistent details [6,12,14], it is much higher than the accuracy of contradictory details [12,13].

Recently, research has shown that the consistency of recall is influenced by the type of interview used at the first recall attempt (T1) [14]. Specifically, Hope et al. [14] compared recall performance between two groups who completed either a Self-Administered Interview (SAI) or a free recall (FR) as the T1-interview and underwent a Cognitive Interview one week later (T2). The SAI is based on the Cognitive Interview [15] and adopts some of its memory-enhancing components, such as the mental context reinstatement, and unlike the FR provides ample retrieval support. It was developed to elicit an early comprehensive statement [16]. Recall with the SAI elicited a greater proportion of consistent details, but a smaller proportion of reminiscent details than recall using FR [14]. The proportion of forgotten and contradictory details and the accuracy rates of all different consistency categories did not differ as a function of T1-interview type. The authors explained the results with the finding that at T1, the SAI group reported much more information than the FR group. As a consequence, there may have been less opportunity to report reminiscent details in the SAI relative to the FR group.

Given that reminiscence is a common phenomenon, the question arises how it can be accounted for from a scientific perspective. Drawing on cognitive theory of memory, Fisher, Brewer, and Mitchell [17] argue that reminiscence may be caused by a change of retrieval cues. This reasoning is based on the well-known principle of varied retrieval [18]. According to this principle, details that cannot be retrieved with one technique may well become accessible with another one. Hence, it is assumed that reminiscence occurs if a retrieval cue is present in the subsequent but not in the first interview. The more disparate the two retrieval attempts the more reminiscence should emerge. According to Fisher et al. [17], the second principle that can account for the emergence of reminiscence is the independence of components. More specifically, complex events consist of many independent components [3,19]. Thus, incorrectly recalling one component should not have an influence on recall of other components. Cognitive theory thus predicts that reminiscence should occur frequently. Moreover, accuracy of reminiscent details should not be low in principle, but among other things depend on the nature of the second interview. Finally, due to the independence of components, any type of inconsistency should be unrelated to overall accuracy.

The question remains as to why laypersons are so skeptical about reminiscence (note that the term layperson is meant in a broad sense throughout the paper, referring to all persons who are not memory experts formally educated in cognitive psychology). In an attempt to capture laypersons’ beliefs about recall consistency, Fisher et al. [17] formulated the courtroom approach of memory. It should be made very clear that this approach is not a scientific theory, but a conglomerate of beliefs assumed to be held by laypersons about memory performance across several retrieval attempts. In the courtroom approach, the central belief is that memories fade over time. Reminiscence constitutes a violation of this principle. As a consequence, laypersons should hold the following assumptions about recall of reminiscent and contradictory statements: 1) Both reminiscent and contradictory details should be inaccurate (relative to consistent details) and indicators of overall inaccuracy. 2) Reminiscence should occur infrequently. 3) Reminiscence should be the result of external influences (e.g., co-witness information). Fisher et al. [17] derived these assumptions from observations of how judges and attorneys behave in the courtroom but did not directly test them. Pointing out that the existing database is “not robust” (p. 133), the authors encouraged further empirical studies to test laypersons’ assumptions summarized in the courtroom approach and extend the existing database on recall consistency.

A literature review yielded one study that directly tested the beliefs summarized in the courtroom approach and compared the assumptions held by laypersons with regards to performance across two recall attempts with actual recall performance. In this study, all participants watched a film about an altercation ([7], Experiment 2). Subsequently, a memory group provided two statements about the content of the film, one immediately and one after a week. A second group of participants who had also seen the film estimated the recall performance of the memory group. This estimation group assumed that overall recall accuracy would decline over time and that the accuracy of reminiscent details would be lower than the accuracy of consistent and forgotten details. However, this did not correspond with the performance of the memory group at all. In fact, actual recall accuracy remained stable over time and the accuracy of reminiscent, consistent and forgotten details did not differ from each other. Moreover, recall accuracy was underestimated invariably. This was especially striking regarding the reminiscent details: While the actually obtained accuracy rate was 84%, estimated performance was as low as 19%.

Even though this study by Oeberst [7] yielded interesting findings and found support for both the existence of the beliefs summarized in the courtroom approach [17] and their discrepancy with reality, it has three important limitations. The first limitation pertains to the ecological validity of the sample that was comprised of students who are unlikely to have in-depth professional experience with obtaining and assessing eyewitness testimony. Hence, it is unclear as to whether the findings transfer to individuals involved in the legal process, such as police detectives. Second, the study did not consider two elements contained in the courtroom approach. Specifically, beliefs about the accuracy of contradictions were not examined. A comparison of the estimated accuracy of contradictory details (of which at least one detail is incorrect) and reminiscent details, however, could yield interesting insights into the relative reliability attributed to reminiscence. Furthermore, while the accuracy rates of consistent, reminiscent and forgotten details were collected in previous research [7], the observed and estimated prevalence of the consistency categories were not. Obtaining this information would be illuminative as to whether the frequency of occurrence of the different consistency categories is over- or underestimated and would also shed light onto the attributed reliability of reminiscence. The third limitation is that the study remained silent concerning potential reasons why reminiscent details are considered incorrect. Knowledge about these reasons could serve as the starting point to revise the content of police training courses so as to increase knowledge on the principles of human memory and to reduce potential bias against reminiscence.

It was the aim of the present study to test the beliefs regarding recall performance over time and consistency of recall that are summarized in the courtroom approach of memory [17] and to test whether experienced police detectives hold these beliefs. As in previous research [7], this was done by contrasting estimates of recall performance with actual recall performance across two recall attempts. More specifically, we sought to extend the findings of Oeberst’s [7] study and to address the limitations of the study, using the following means. First, instead of a student sample, we relied on an ecologically valid sample of experienced police detectives as the estimation group. Second, to test the assumptions about the frequency of reminiscence, which have not been considered in previous research, we collected estimates of the frequencies of the different consistency categories in addition to the estimates of their accuracy rates. Third, going beyond documenting the discrepancies between laypersons’ beliefs and actual recall performance, we for the first time explicitly inquired about reasons for the mistrust in reminiscence. Fourth, we considered the recent finding that consistency rates vary as function of T1-interview type [14], because this may influence the extent of the discrepancy between the beliefs and actual recall performance. Therefore, we varied the T1-interview type (SAI vs. FR) both in the memory and in the estimation group.

Based on the beliefs summarized in the courtroom approach and previous findings [7], we hypothesized that recall accuracy would be underestimated without exception. Second, we expected an interaction such that the estimated decrease of accuracy over time would be stronger than actual decrease of accuracy. Third, the discrepancy between the accuracy of reminiscent details and the accuracy of consistent and forgotten details should be greater in the estimation than in the memory group. Fourth, the discrepancy between the accuracy of reminiscent and contradictory details should be smaller in the estimation than in the memory group. Finally, it was hypothesized that the proportion of consistent and forgotten details should be overestimated, and the proportion of reminiscent and contradictory details underestimated.

## Method

### Ethics Statement

This study was approved by the ethics committee of the Faculty of Psychology and Neuroscience of Maastricht University and follows the rules stated in the Declaration of Helsinki. All participants read and signed a written informed consent.

### Participants and Design

The memory group consisted of *N* = 84 German police students (22 women; *n* = 23 members of the criminal police and *n* = 61 of the uniformed police; age 20 to 39, *M* = 24.6, *Mdn* = 23 years). None of the participants of the memory group indicated having previous knowledge of or practical experience with the SAI (note that the SAI was neither part of the students’ training nor in use by this German police force at the time of testing).

The estimation group consisted of *N* = 93 detectives of the Dutch police. Twelve participants of the estimation group had to be excluded because they were shown a wrong film version, leaving *N* = 81 for analysis (26 women; age 26 to 58, *M* = 42.2, *Mdn* = 42 years) with an average work experience of *M* = 19.7 years (*SD* = 9.4; range 5 to 38 years) and an average experience of *M* = 12.8 years (*SD* = 8.9; range 0 to 38 years) in interviewing eyewitnesses. Of these, 35% had heard of the SAI prior to the study, mostly from interview courses (75%) or colleagues (18%), but no one had used it. Participation occurred on a voluntary basis.

**Fig 1:**
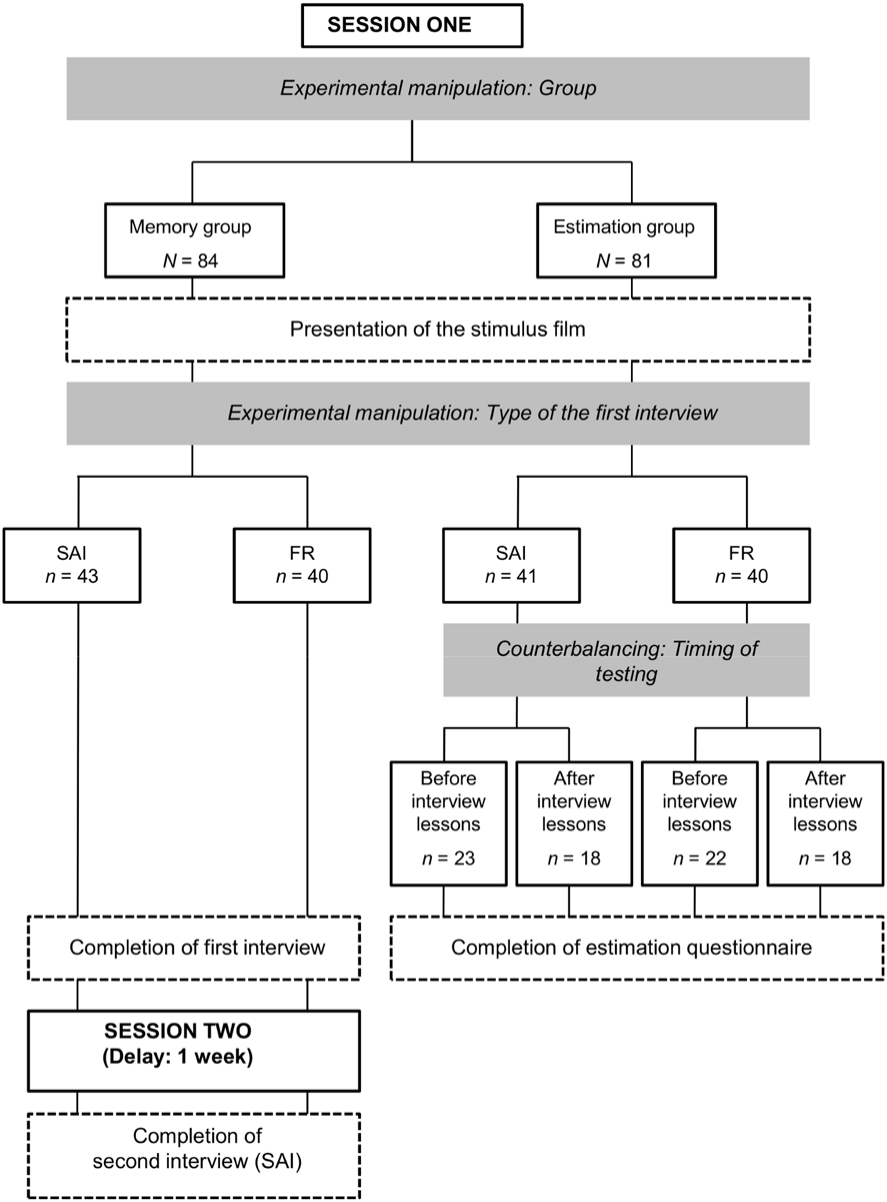
Design and procedure of the study.

The study employed a 2 (group: memory vs. estimation) x 2 (interview type at the first recall attempt: SAI vs. FR) between-participants design. The dependent variables were overall accuracy at T1 and T2, accuracy and proportion of details mentioned at T1 but forgotten at T2, as well as accuracy and proportion of consistent, reminiscent and contradictory details at T2. Fig. 1 provides an overview of the design.

### Materials

**Film**. To test an unrelated research question, we created two different film versions (A and B) from the same video footage that showed the non-violent theft of a wallet. The films were identical apart from a few short sequences that were shown in one film version, but were removed in the other version and vice versa (e.g., thief and accomplice point to the victim or thief disposes of the emptied wallet). Note, however, that the act of stealing was present in both versions. Both film versions were edited to last approximately 2:10 min. In both films, the female victim chats with two friends (male and female) and is observed from a distance by the thief and the accomplice (both male). After the friends have left the scene, the accomplice approaches and distracts the victim by pretending to ask for directions. At the same time, the thief steals the wallet from her purse and subsequently both perpetrators leave the scene. The two friends return and the victim realizes that her wallet is missing. In a last scene, the thief is shown inspecting the content of the wallet.

**Interviews used in the memory group: Self-Administered Interview**. In the SAI ([16]; see Hope, Gabbert, and Fisher [20] for a more detailed description), participants first mentally reinstated the context by thinking back to the incident, picturing in their minds what they saw and recreating the thoughts and emotions they had at the time. Supported by non-leading recall cues, in separate sections, they provided descriptions of the course of events, the appearance of the perpetrator(s), and, if applicable, of potential other witnesses or vehicles involved. To promote multiple and varied retrieval, participants were also requested to draw a sketch of the scene, so as to facilitate the retrieval of spatial information. In a final section, the witnessing conditions were prompted (e.g., What time of day did the event occur?). Throughout the interview, participants were instructed to provide the most complete and accurate account of the witnessed incident possible, but not to guess.

**Interviews used in the memory group: Free recall**. The FR comprised only one retrieval attempt and requested participants to report all details they could remember about the sequence of actions and events, and of all persons involved, including the perpetrator(s) and other witnesses (see Gabbert et al. [16]). Analogous to the SAI, participants were instructed to provide the most complete and accurate account possible, but were discouraged from guessing.

**Questionnaire used in the estimation group**. In the estimation questionnaire, participants of the estimation group were informed that another group (i.e., the memory group) had also watched the same film and that it would be their task to estimate the recall performance of this group. No information was disclosed as to who the memory group was to prevent any potential biases. Participants were informed that the interview took place directly after the film and were provided with a description of either the SAI or FR, depending on the T1-interview condition. This description was supposed to acquaint participants with the respective interview, including its instructions and structure. Participants were then requested to estimate the overall accuracy obtained in the interview by the memory group at T1. For this purpose, the term accuracy and how accuracy is calculated were explained in simple terms. After providing this estimate, participants were informed that one week later there was a second interview about the film in the form of the SAI. For the participants in the FR condition, the description of the SAI was provided at this point. Subsequently, participants estimated the accuracy and the proportion of the details that were mentioned at T1 but forgotten at T2, the overall accuracy and the accuracy and proportion of the consistent, reminiscent and contradictory details obtained in the second interview. For the sake of comprehensibility, examples of all consistency categories were provided. Participants were also asked to briefly explain in their own words why they regarded reminiscent details as accurate or inaccurate, respectively.

### Procedure

**Memory group**. The participants of the memory group were tested in groups of up to 20 people (i.e., one class of students) during their lessons at the university. They were tested by a dyad of experimenters, of which only one was formally blind. Although it is suboptimal that not both experimenters were blind, the following measures were taken to avoid an influence on participants. First, all instructions were provided in writing. Second, if questions occurred, they were answered by the blind experimenter (i.e., the interaction between participants and the non-blind experimenter was kept to a minimum). Third, if participants asked about what they should recall, they were simply reminded to recall as completely and accurately as possible, as requested in the instructions. In the first session, participants were informed that as part of an eyewitness experiment they would view a film and subsequently answer some questions about it. After signing the informed consent and providing demographic information, participants of each class watched either film version A or B determined by random assignment. After a delay of about 30 minutes during which the lesson was continued, participants completed either an SAI or an FR. They were informed that there would be a second session one week later. However, nothing was disclosed about the content of this session. In this second session, all participants filled in an SAI as the T2-interview. Finally, participants received a recognition test to address an unrelated research question. To control for group dynamics, in both sessions, individual seating was ensured and, to prevent a motivation loss among the slower participants, the group was only dismissed after the last student had finished. Furthermore, participants were instructed to complete all materials alone (i.e., without the help of others) and reminded not to talk with others, including fellow participants, about the experiment. Finally, the experimenters stayed in the room during testing to immediately cut off any potential emerging communication between participants (note, however, that this was hardly necessary).

**Estimation group**. The participants of the estimation group were also tested in groups. Analogous measures to control for group dynamics as in the memory group were applied. The experimenter in the estimation group was blind to the hypotheses. Hence, it can be ruled out that any expectations regarding the estimations were communicated to participants. Testing occurred in the framework of an extensive course offered to detectives by the Dutch police academy on how to investigate comprehensive cases. This course consisted of several units one of which dealt with interviewing witnesses and the reliability of witness accounts. Note that the course covered the SAI, but not the issue of repeated interviewing and reminiscence. Timing of testing was counterbalanced so that half of the classes were tested before and half after the lessons on interviewing witnesses (see Fig. 1). Participants were informed that they would take part in an experiment on eyewitness accounts and that they would watch a short film which would be followed by a questionnaire. After signing the informed consent and providing demographic information, they were shown film A (Only film A was used for the estimation group, because the two film versions were considered to be equivalent. Note that within the memory group, there were no significant main effects of film on any of the dependent variables. For T2-accuracy and the proportion of forgotten details, there were significant Film by T1-interview interactions. However, as there was no apparent reason or theoretical rationale for the interactions to occur, we decided not to consider the film version in the estimation group.). Subsequently, the participants completed one of two versions of the estimation questionnaire (i.e., either the version describing the SAI as the first interview or the version describing the FR as the first interview). Upon completion of data collection all participants were fully debriefed.

### Coding

The statements of the memory group were transcribed and coded employing a comprehensive coding scheme consisting of 685 details, of which approximately 56% were person, 19% setting, 13% action and 12% object details. The statement “the thief (1) sat (2) at the rightmost (3) table (4)” would yield four details (see Sauerland, Krix, van Kan, Glunz, and Sak [21], for a similar approach). Subjective responses, such as “he was ugly”, were not scored. A detail was considered correct, if it matched the content of the film, and considered incorrect, if it did not match the content of the film. Details were considered confabulated when they were both incorrect and non-existent (e.g., recalling a hat when no headgear was worn; see Sauerland et al. [21]).

Details were also coded for consistency. Specifically, consistent details were recalled in both interviews (e.g., describing the thief as blonde in both interviews). Details mentioned in the second interview that were in direct conflict with details mentioned in the first interview were considered contradictory (e.g., describing the hair color as brown in the second and as blonde in the first interview). Note that for determining the accuracy of contradictions, the detail reported in the second interview was used. Forgotten details were only reported in the first but not the second interview and reminiscent details were only reported in the second interview.

**Inter-coder reliability**. To establish inter-coder reliability, the randomly selected statements of 15 participants from the memory group were independently coded by two coders. Due to the elaborate and unambiguous coding scheme and the fact that coders were trained in using the scheme, inter-coder reliability was high. Specifically, across consistency categories and accuracy, Cohen’s κ was 0.88, p < .001, indicating almost perfect agreement [22].

## Results

An *alpha* level of .05 was used for all statistical tests. We report Cohen’s *d* [23] for dependent or independent samples for main effects with *df* = 1 in the numerator and *η*_*p*_ *²* for main effects with *df* > 1 and interaction effects [24]. When the estimates of the estimation group did not differ as a function of timing of testing (before vs. after the eyewitness lessons), *p*s ≥ .168, they were collapsed across timing. Otherwise, analyses were run separately for the two subgroups. When the dependent variables did not differ as a function of T1-interview type, *p*s ≥ .108, T1-interview type was removed and the analyses rerun.

### Accuracy

Table 1 displays the means and standard deviations of T1 and T2 recall accuracy, as well as the accuracy of the different consistency categories of the memory group and the corresponding estimates of the estimation group.

**Table 1:** Accuracy of overall T1 and T2 recall and of the consistency categories for the memory group and the corresponding estimates of the estimation group.

	Memory group		Estimation group	
	*M (%)*	*SD*	*M (%)*	*SD*
Overall T1	87.47	4.70	46.19^c^	18.31
Overall T2	85.97	4.81	27.10^c^	16.36
Consistent	91.96^ab^	3.61	55.00^de^	21.85
Forgotten	86.16^a^	8.01	37.90^de^	20.14
Reminiscent	85.53^b^	8.32	28.59^d^	19.86
Contradictory	39.87^ab^	30.48	25.34^e^	15.17

Means in a column sharing the same superscript differ at *p* < .05.

**Recall performance over time**. To test whether the courses of actual and estimated accuracy rates over time differed, we calculated mixed-model ANOVAs with group (memory vs. estimation) and recall attempt (T1 vs. T2) as the independent variables. Accuracy at T1 and T2 were the dependent variables. The significant main effects of group, F(1, 163) = 781.06, p < .001, d = 4.35, and time, F(1, 163) = 161.77, p < .001, d = 0.31, were qualified by a significant interaction, F(1, 163) = 117.89, p < .001, η²_p_ = 0.42. As expected, the analysis of the simple main effects revealed that within the memory group, accuracy remained stable from T1 (M = 87.47%, SD = 4.70) to T2 (M = 85.97%, SD = 4.81), F(1, 163) = 1.76, p = .186, d = 0.32. In contrast, the estimation group assumed that accuracy would sharply decline from T1 (M = 46.19%, SD = 18.31) to T2 (M = 27.10%, SD = 16.36), F(1, 163) = 272.96, p < .001, d = 1.09. Note that, also as expected, the accuracy of the memory group was significantly underestimated both at T1, F(1, 163) = 399.90, p < .001, d = -3.11, and at T2, F(1, 163) = 998.84, p < .001, d = -4.92.

**Consistency categories**. To analyze whether the accuracy rates of the consistency categories differed within the memory and the estimation group, we calculated mixed-model ANOVAs with group (memory vs. estimation) and consistency category as the independent variables. Accuracy of the four consistency categories were the dependent variables. The significant main effects of group, F(1, 156) = 502.77, p < .001, d = 3.58, and consistency category, F(3, 468) = 175.70, p < .001, η²_p_ = 0.53, were qualified by a significant interaction, F(3, 468) = 49.94, p < .001, η²_p_ = 0.24. Post-hoc analyses with Bonferroni tests (as they yield adjusted significance levels, the usual level of .05 to determine statistical significance can be applied) showed that within the memory group, accuracy of the consistent details (M = 91.96%, SD = 3.61) was significantly higher than accuracy of the forgotten (M = 86.16%, SD = 8.01), p = .024, d = 0.89, reminiscent (M = 85.53%, SD = 8.32), p = .011, d = 1.03, and contradictory details (M = 39.87%, SD = 30.48), p < .001, d = 2.54. Both forgotten, p < .001, d = 2.07, and reminiscent details, p < .001, d = 2.12, were more accurate than the contradictory details. Accuracy of forgotten and reminiscent details did not differ, p = .999, d = 0.12.

Within the estimation group and analogous to the memory group, estimated accuracy of the consistent details (*M* = 55.00%, *SD* = 21.85) was significantly higher than estimated accuracy of the forgotten (*M* = 37.90%, *SD* = 20.14), *p* < .001, *d* = 0.79, reminiscent (*M* = 28.59%, *SD* = 19.86), *p* < .001, *d* = 1.26, and contradictory details (*M* = 25.34%, *SD* = 15.17), *p* < .001, *d* = 1.53. However, in contrast to the results found in the memory group, estimated accuracy of the forgotten details was higher than estimated accuracy of both reminiscent, *p* < .001, *d* = 0.47, and contradictory details, *p* < .001, *d* = 0.70. Interestingly, the estimated accuracy of the reminiscent and contradictory details did not differ, *p* = .999, *d* = 0.18.

Also in line with our hypotheses, the actual accuracy of all consistency categories was underestimated (consistent: *F*[1, 163] = 233.73, *p* < .001, *d* = -2.38; forgotten: *F*[1, 162] = 413.57, *p* < .001, *d* = -3.18; reminiscent: *F*[1, 163] = 584.33, *p* < .001, *d* = -3.76; contradictory: *F*[1, 157] = 14.55, *p* < .001, *d* = -0.60).

To summarize the results regarding actual and estimated accuracy of recall, all our hypotheses were confirmed. First, recall accuracy was underestimated without exception. Second, while actual accuracy remained stable from T1 to T2, a sharp decline was assumed by the estimation group. Third, the estimation group expected that reminiscent details would be as inaccurate as contradictory details and much less accurate than consistent details. In reality, even though consistent details were most accurate, accuracy of reminiscent details was at a high level and a lot higher than accuracy of contradictions. The results are in line with previous findings [7] and confirm the existence of the beliefs summarized in the courtroom approach of memory [17] that do not correspond with actual recall performance. In the following, the results on the proportion of the consistency categories will be reported.

### Proportion of the Different Consistency Categories

To compare the estimated and actual proportions of the consistency categories, we calculated one-way ANOVAs with group (memory vs. estimation) or two-way ANOVAs with group and T1-interview type as the independent variables. For *n* = 13 participants the sum of the proportions of consistent, reminiscent and contradictory details of all details recalled at T2 was unequal to 100% (although participants were reminded to provide estimates that add up to 100%), indicating that they had misunderstood the question. Hence, they were excluded from analysis. For consistent and contradictory details, timing of the questionnaire had an influence. Specifically, in the group that had not yet followed the eyewitness lessons (*M*_*consistent*_ = 60.08%, *SD*_*consistent*_ = 16.53; *M*_*contradictory*_ = 18.92%, *SD*_*contradictory*_ = 11.64), the estimated proportion of consistent details was higher, *F*(1, 63) = 5.65, *p* = .020, *d* = 0.59, and the estimated proportion of contradictory statements lower, *F*(1, 63) = 8.81, *p* = .004, *d* = -0.74, than in the group who had already followed the lessons (*M*_*consistent*_ = 49.64%, *SD*_*consistent*_ = 19.19; *M*_*contradictory*_ = 30.00%, *SD*_*contradictory*_ = 18.56). Hence, for the analyses of these variables the estimation group was split.

**Forgotten details**. The proportion of T1-information that was forgotten at T2 was significantly overestimated (estimation group: M = 42.85%, SD = 20.91; memory group: M = 24.20%, SD = 8.80), F(1, 163) = 56.48, p < .001, d = 1.17.

**Consistent details**. When the memory group was compared to the participants of the estimation group who had already received the eyewitness interview lessons, both main effects (group: F[1, 108] = 52.55, p < .001, d = 1.40; interview type: F[1, 108] = 7.70, p = .007, d = 0.77) and the interaction, F(1, 108) = 9.24, p = .003, η²_p_ = 0.08, were significant. The proportion of consistent information was underestimated both when the T1-interview was an SAI (estimation group: M = 49.29%, SD = 21.29; memory group: M = 76.94%, SD = 8.60), F(1, 108) = 53.25, p < .001, d = -2.16, and when it was an FR, although to a lesser degree (estimation group: M = 50.00%, SD = 17.65; memory group: M = 61.31%, SD = 9.13), F(1, 108) = 8.81, p = .004, d = -0.96. When the memory group was compared to the participants of the estimation group who had not yet received the eyewitness interview lessons, analogous results were obtained with one exception. Specifically, when the T1-interview was an FR, actual and estimated proportion of consistent details did not differ (estimation group: M = 62.89%, SD = 12.40; memory group: M = 61.31%, SD = 9.13), F(1, 119) = 0.23, p = .629, d = 0.15. Table 2 displays the means and standard deviations of the actual and estimated proportion of consistent details.

**Table 2:** Proportion of consistent details as a function of T1-interview type and group.

	Memory group		Estimation group before eyewitness lessons		Estimation group after eyewitness lessons	
Type of T1-interview	*M (%)*	*SD*	*M (%)*	*SD*	*M (%)*	*SD*
SAI	76.94^ab^	8.60	57.40^a^	19.64	49.29^b^	21.29
FR	61.31^c^	9.13	62.89	12.40	50.00^c^	17.65
Total	69.31	11.80	60.08	16.53	49.64	19.19

The estimation group is split as a function of whether they had already followed the eyewitness lessons. Means in a row sharing the same superscript differ at *p* < .05.

**Reminiscent details**. For the proportion of reminiscent details, both main effects (group: F[1, 147] = 16.99, p < .001, d = 0.56; interview type: F[1, 147] = 18.69, p < .001, d = 0.70) and the interaction, F(1, 147) = 32.93, p < .001, η²_p_ = 0.18, were significant. Analysis of the simple main effects revealed that when the first interview was an SAI, the actual and estimated proportions of reminiscent details did not differ (estimation group: M = 21.88%, SD = 13.37; memory group: M = 19.21%, SD = 8.24), F(1, 147) = 1.33, p = .250, d = 0.25. However, when the first interview was an FR, the proportion of reminiscent details was significantly underestimated (estimation group: M = 19.55%, SD = 8.69; memory group: M = 35.82%, SD = 9.70), F(1, 147) = 47.72, p < .001, d = -1.75. Table 3 displays the means and standard deviations of the actual and estimated proportion of reminiscent details.

**Table 3:** Proportion of reminiscent details as a function of T1-interview type and group.

	Memory group		Estimation group	
Type of T1-interview	*M (%)*	*SD*	*M (%)*	*SD*
SAI	19.21	8.24	21.88	13.37
FR	35.82^a^	9.70	19.55^a^	8.69
Total	27.32	12.22	20.73	11.28

Means in a row sharing the same superscript differ at *p* < .05.

**Contradictory details**. The proportion of contradictory details found in the memory group (M = 3.37%, SD = 2.20) was significantly overestimated both by the participants of the estimation group who had already followed the eyewitness interview lessons (M = 30.00%, SD = 18.56), F(1, 108) = 166.35, p < .001, d = 2.84, and by those who had not yet followed the eyewitness lessons (M = 18.92%, SD = 11.64), F(1, 119) = 139.80, p < .001, d = 2.30. All other main effects and interactions were non-significant, Fs ≤ 1.78, ps ≥ .185, ds ≤ 0.15, η²_p_s < 0.01.

To summarize, we found mixed support for our hypotheses regarding the proportions of the consistency categories. As expected, the amount of forgetting was overestimated. The frequency of reminiscence was underestimated only when the T1-interview was an FR, but not when it was an SAI, showing that it may be important to take into consideration the T1-interview type when examining laypersons’ beliefs. This result was due to a higher reminiscence rate in the FR memory group, as previously found by Hope et al. [14]. In direct contrast to our hypotheses, the amount of consistency was underestimated and the amount of contradictions overestimated.

### Reasons for High or Low Accuracy Rates of Reminiscent Details

The reasons provided by the estimation group as to why they believe that reminiscent details are characterized by high or low accuracy rates, respectively, can be found in Table 4 (note that some participants provided several reasons). The majority of the responses reflect a skeptical attitude towards reminiscent details, mostly out of concern that they have been contaminated by external influence. We will address the responses in more detail in the discussion.

**Table 4:** Reasons provided by the estimation group for a low or high accuracy of reminiscent details.

**Reasons provided for a low accuracy of reminiscent details**	% (*n*)
New details are the result of compromising external influence (including media reports, co-witness discussions and information leaked by the police)	48.1 (39)
The witness fills in gaps (also to make the story more coherent)	13.6 (11)
Memory decreases over time	9.9 (8)
All stored details can already be retrieved in the first interview	3.7 (3)
Replaying the incident in one’s mind distorts the mental picture of the incident	3.7 (3)
The witness makes details up	2.5 (2)
Witnesses do not change their stories	2.5 (2)
The witness just wants to please the interviewing police officer	2.5 (2)
Witnesses have a bad memory in general	1.2 (1)
The recollection during the second interview is based on what was said in the first interview and not on the actual incident	1.2 (1)
Reminiscent details are the result of a source memory error	1.2 (1)

**Reasons provided for a high accuracy of reminiscent details**	
Replaying the incident in one’s mind can elicit relatively accurate reminiscent details	9.9 (8)
The stress is reduced in the second interview	3.7 (3)
Reminiscent details can be quite accurate if decent interview techniques/good questions are used	2.5 (2)
The witness remembered the detail all along, but did not consider it important at first	1.2 (1)
Reminiscent details have not been influenced by external factors	1.2 (1)
Reminiscent details have been influenced by external factors	1.2 (1)
Not all details are accessible in the first interview	1.2 (1)

### Additional Analyses

In an additional analysis, we examined whether inconsistencies in recall were related to overall accuracy within the memory group. The number of reminiscent details was not related to T2-recall accuracy, *r*(82) = -.03, *p* = .793. However, there was a large negative correlation between number of contradictions and T2-recall accuracy, *r*(82) = -.50, *p* < .001. Members of the legal system have been found to use the number of contradictions to challenge the accuracy of the *rest* of the statement (i.e., excluding contradictions; Fisher et al. [17]). Hence, the correlation between the number of contradictions and accuracy of the rest of the statement may be more relevant. This correlation was lower, but still of moderate size, *r*(82) = -.32, *p* = .003.

In contrast to the belief of the estimation group, accuracy of recall did not decline over time in the memory group (see above). It may be the case, however, that in line with a quantity-accuracy trade-off [25], the deterioration of memory manifested itself in reduced recall quantity rather than in reduced accuracy. To test this idea, the quantity of recall of the memory group was analyzed as a function of T1-interview type (SAI vs. FR) and recall attempt (T1 vs. T2). Comparing actual recall performance and estimations is not possible for this variable because the quantity of recall was not collected in the estimation group. The main effect of T1-interview type was non-significant, *F*(1, 82) = 1.54, *p* = .219, *d* = 0.27. The significant main effect of time, *F*(1, 82) = 6.57, *p* = .012, *d* = 0.15, was qualified by a significant Time by T1-Interview Type interaction, *F*(1, 82) = 67.25, *p* < .001, *η²*_*p*_ = 0.45. The simple main effects analyses revealed that within the SAI-group, the quantity indeed significantly decreased from T1 (*M* = 130.67, *SD* = 28.47) to T2 (*M* = 119.54, *SD* = 30.12), *F*(1, 82) = 16.28, *p* < .001, *d* = -0.38. In contrast, within the FR-group, the quantity significantly increased from T1 (*M* = 106.44, *SD* = 31.72) to T2 (*M* = 127.71, *SD* = 33.80), *F*(1, 82) = 56.58, *p* < .001, *d* = 0.65.

## Discussion

In the present study, we compared police detectives’ estimates of eyewitnesses’ performance across two recall attempts with actual recall performance. Thereby, the present study aimed to test layperson’s beliefs about the consistency of recall that are summarized in the courtroom approach of memory [17] and to test whether experienced police detectives hold these beliefs. In doing so, we extended earlier findings in this field and addressed the limitations of previous research [7] by use of the following means. First, to our knowledge, this is the first study that directly compared frequency estimates with actual frequencies of the consistency categories. Second, for the estimations, we relied on an ecologically valid sample of police detectives who had on average 19 years of work experience and 13 years of investigative interviewing experience. Their estimates were compared to the recall performance of police students who only recently started their career at the police and are therefore unlikely to differ from non-police witnesses. Note that the police students’ recall performance was indeed comparable to the performance of non-police witnesses in other experiments conducted in our lab that used the same interview types [26]. Third, taking into account recent findings [14], the interview type at T1 was varied, as this may have an impact on the discrepancy between the beliefs and actual recall performance. Fourth, by for the first time explicitly inquiring about the reasons for the mistrust in reminiscence, this study went beyond documenting discrepancies between layperson’s beliefs and witnesses’ actual recall performance.

Before addressing the results on the beliefs, a general note on reminiscence as a result of repeated interviews seems in order. Like many previous studies, the present study identified reminiscence as a common phenomenon [6,9,12,14]. Every participant of the memory group experienced reminiscence. Moreover, the amount of reminiscence was no significant indicator of overall accuracy, showing that its occurrence should not serve to discredit witnesses in court (note that this is different for the amount of contradictions which was negatively related to accuracy of the rest of the testimony). Rather, the present findings support the notion that repeated interviews can be a means to improve recall performance, as they present the opportunity to report new, that is, reminiscent details at a reasonably high accuracy rate [13,14,27]. Moreover, they illustrate the importance to not indiscriminately use the term inconsistency but to differentiate between contradictions and reminiscence [7,17].

Turning to the results on the beliefs, recall that the courtroom approach summarizes beliefs about recall consistency that are supposedly held by laypersons [17]. Based on the assumption of fading memory over time, laypersons should assume both reminiscent and contradictory details to be inaccurate and indicators of overall inaccuracy. Moreover, according to laypersons, reminiscence should occur infrequently and be the result of external influences (e.g., co-witness information). Indeed, the present study revealed that experienced police detectives hold these implicit beliefs that do not correspond with actual recall performance. In the following, we will first discuss the results regarding accuracy, followed by the results regarding the frequencies.

In agreement with the belief of memory fading over time, the estimation group assumed a sharp decline of accuracy, whereas it actually remained stable. Note, however, that this discrepancy does not preclude actual memory fading. The estimation group may have erroneously assumed that memory degradation inevitably leads to reduced accuracy. Yet, from research dealing with the metamemory model by Koriat, Goldsmith, and colleagues it is known that memory degradation may manifest itself in reduced quantity [25] or reduced level of specificity [28,29], while accuracy remains stable [30]. A decrease of quantity from T1 to T2 was observed in the memory group, at least for those who completed an SAI at T1. In any case, it can be stated that concerns about an excessive decline of accuracy seem unwarranted.

The detectives also displayed a considerable amount of mistrust in the accuracy of reminiscence as predicted in the courtroom approach of memory [17]. Specifically, the estimation group assumed that reminiscent details would be as inaccurate as contradictory details. This is striking because the probability of a contradiction at T2 being correct cannot exceed 50%. In reality, even though consistent details were most accurate, accuracy of reminiscent details was high and much higher than accuracy of contradictions, as found previously [6,12,13].

Overall, the results regarding estimated and actual accuracy are analogous to those obtained by Oeberst [7]. Thus, it seems that experienced police detectives hold similar implicit beliefs about memory performance across two retrieval attempts and exhibit the same mistrust in reminiscence as students who are unlikely to have in-depth experience with obtaining and assessing eyewitness testimony. As in Oeberst’s [7] study, the accuracy rates were always underestimated. This general pattern of underestimation is difficult to reconcile with the notion that police officers usually consider eyewitness evidence to be crucial and to frequently provide critical investigation leads [31]. Regarding overall accuracy, the detectives seemed to be more skeptical than students, as their estimates were even lower than those provided by a student sample [7]. Interestingly, estimated accuracy of consistent and reminiscent details received similar estimates by both samples.

As to the frequencies of the consistency categories, the amount of forgetting was overestimated, which is in line with our hypotheses. Reflecting the belief that reminiscence is uncommon [17], the frequency of reminiscence was underestimated in the FR group, while this was not the case in the SAI group. The pattern emerged not because the beliefs of the estimation group differed across interview types (the estimates of the frequency of reminiscence were approximately the same for FR and SAI). Rather, as reported previously [14], the actual reminiscence rate was much higher in the FR than in the SAI group. This shows that it is important to consider the type of T1-interview when examining beliefs about recall consistency. Also unexpectedly, due to the dramatic overestimation of the frequency of contradictions, the frequency of consistent recall was underestimated. The latter also reflects skepticism towards eyewitness accounts in the sample of detectives. The eyewitness lessons made the detectives even more critical as indicated by the larger discrepancy of the estimates in the subgroup that had already followed the lessons.

Turning to the reasons provided for an assumed high or low accuracy of reminiscence, it was most frequently stated that reminiscence was the product of negative external influences, such as co-witness discussions or media coverage. This reflects the belief that reminiscence is the result of an unusual non-cognitive mechanism, which would be expected by the courtroom approach [17]. In addition, alleged cognitive mechanisms were specified, such as an assumed regular tendency among witnesses to fill in gaps, the ability to exhaustively recall all details in the first interview and a decrease of memory over time. As Erdelyi [9] pointed out, however, forgetting does not preclude reminiscence. It should be noted, though, that there were a few detectives who acknowledged that reminiscent details can be quite accurate. Yet, only one specified that not all details may be accessible in the first interview and two participants acknowledged that reminiscence may be related to the kind of questioning of the T2-interview, but did not explicate how. The reasons identified in the present study provide important insights into the beliefs and misconceptions held by police detectives about human memory functioning and reveal substantial knowledge gaps in this field. As such, they provide leads for the content of police detectives’ trainings. These will be addressed below.

Turning to potential limitations of the study, it should be noted that the estimation group provided the estimates of the first and second recall attempt during a single session and not in two sessions with a one week interval as the memory group. However, we do not believe that this poses a threat to the validity of the results. Specifically, Oeberst [7] (Experiment 2) included two estimation groups, one that provided all estimates in one session and one that provided the estimates at the same time as the memory group. Apart from one dependent variable, there were no differences in results between the two estimation groups. Hence, it is not decisive whether the estimation group actually experienced the retention interval. Another limitation may pertain to the procedure that before providing the estimates, the estimation group watched the stimulus film. This was done to give the estimation group a better idea of the witnessing conditions and hence a chance to correctly assess the performance of the memory group. Still, they failed to correctly predict recall performance. Our approach differs from real cases where police officers do not have the opportunity to see the crime prior to assessing the witnesses’ statement. However, if the estimation group had not been informed about the witnessing conditions, it could have been argued that the discrepancies between estimated and actual performance emerged because the estimation group had no clue what to assess. Notwithstanding, we do not believe that this deviation from real cases influenced the findings, because Oeberst [7] obtained similar results across two experiments, irrespective of whether the estimation group had inspected the to-be-remembered material or not. Finally, descriptions were used to inform the estimation group about the structure of the interview tools. It would have been more realistic if they had received copies of the interviews. Yet, it is unclear and remains an empirical question for future studies whether this influenced our results.

Even though the detectives examined in the present study were right in principle to judge reminiscent details as less accurate than consistent details and to point out the potential deleterious influence of incorrect post-event information, this should not give rise to a general mistrust in reminiscence or overgeneralization, respectively. Note that overgeneralization is also apparent in other areas of eyewitness memory, such as in the supposition that child eyewitnesses are generally less credible than adults, while recent research has shown that in some cases, children’s recollections may be *less* vulnerable to suggestive influence than adults’ recollections (see Brainerd [32], for an overview). Such misjudgments may lead to unjustified discrediting of witnesses. This is unfortunate given the central role of the police for gathering evidence. More specifically, if the police are not convinced of the reliability of witnesses’ statements because of inconsistencies, they are likely not to invest additional resources into the investigation or may not follow up on certain details mentioned by the witness. Ultimately, this may jeopardize the success of the investigations. This demonstrates the importance to revise trainings for members of the legal system regarding the functioning of human memory.

It is clear that police officers are but one of several occupational groups in the legal system concerned with assessing the accuracy of eyewitness statements. We therefore encourage replication with other groups, that is, lawyers and judges. Still, this study is also of relevance for these groups. During cross-examination, reminiscent information should be distinguished from direct contradictions. Reminiscent details should not be used to question the accuracy of the whole statement, but be taken seriously, because chances are high that they are accurately recalled details.

From the present findings recommendations for police officers’ trainings on the structure of human memory and interviewing eyewitnesses can be derived. While the course in which the estimation group took part covered network models of memory [33], our results suggest that this may not be sufficient to counteract mistrust in reminiscence. First, the trainings should foster a differentiated view on eyewitnesses’ performance to prevent the emergence of overgeneralization. Second, a greater emphasis should be put on teaching the structure of human memory. Building on the network models, courses should in addition deal with the principle of the independence of components of a complex event [17] and the notion that multiple and varied retrieval attempts make different aspects of the memory accessible [18]. Discussing these two memory principles should illustrate that reminiscence is a normal phenomenon which occurs concurrent with forgetting [9] and is largely independent of overall accuracy. In this regard, the importance of differentiating between reminiscence and contradictions should be made clear. Moreover, to account for reminiscence in a given eyewitness account, it is important to convey strategies how to inquire whether the witness may have been exposed to post-event information and whether the interviews conducted provided different retrieval cues. As our findings have shown, memory decay does not necessarily manifest itself in reduced accuracy over time. Instead, the quantity or the level of precision may deteriorate while accuracy remains stable. Presenting the metamemory framework by Koriat and Goldsmith [25,28,29] that provides an explanation for these trade-offs between quantity or level of precision, respectively, and accuracy would be important to illustrate this. Finally and perhaps more importantly, reminiscence should not only be dealt with in the theoretical elements of the courses, but also in the practical elements. Specifically, in addition to improving participants’ interview skills practice interviews, which are a routine part of interview courses, could be used for teaching the concept of reminiscence. That is, the participants could interview each other on several occasions about the same incident and in the process directly experience reminiscence themselves. Such a personal experience may increase comprehension and acceptance of reminiscence. For trainings that already do cover reminiscence, we encourage checking whether they include the above mentioned principles. Incorporating these principles into police training courses may help reduce the bias against reminiscence and subsequent misjudgments of the witnesses’ credibility. Ultimately, this may lead to more accurate decisions in court.
